# Long-Lived Plasma Cells in Mice and Men

**DOI:** 10.3389/fimmu.2018.02673

**Published:** 2018-11-16

**Authors:** Siggeir F. Brynjolfsson, Linn Persson Berg, Teresa Olsen Ekerhult, Inga Rimkute, Mary-Jo Wick, Inga-Lill Mårtensson, Ola Grimsholm

**Affiliations:** ^1^Department of Microbiology and Immunology, Institute of Biomedicine, University of Gothenburg, Gothenburg, Sweden; ^2^Department of Infectious Diseases, Institute of Biomedicine, University of Gothenburg, Gothenburg, Sweden; ^3^Department of Urology, Institute of Clinical Sciences, University of Gothenburg, Gothenburg, Sweden; ^4^Department of Rheumatology and Inflammation Research, Institute of Medicine, University of Gothenburg, Gothenburg, Sweden; ^5^B Cell Physiopathology Unit, Immunology Research Area, Bambino Gesù Children's Hospital IRCCS, Rome, Italy

**Keywords:** long-lived plasma cells, B-cells, germinal centers, mice, humans

## Abstract

Even though more than 30 years have passed since the eradication of smallpox, high titers of smallpox-specific antibodies are still detected in the blood of subjects vaccinated in childhood. In fact, smallpox-specific antibody levels are maintained in serum for more than 70 years. The generation of life-long immunity against infectious diseases such as smallpox and measles has been thoroughly documented. Although the mechanisms behind high persisting antibody titers in the absence of the causative agent are still unclear, long lived plasma cells (LLPCs) play an important role. Most of the current knowledge on LLPCs is based on experiments performed in mouse models, although the amount of data derived from human studies is increasing. As the results from mouse models are often directly extrapolated to humans, it is important to keep in mind that there are differences. These are not only the obvious such as the life span but there are also anatomical differences, for instance the adiposity of the bone marrow (BM) where LLPCs reside. Whether these differences have an effect on the function of the immune system, and in particular on LLPCs, are still unknown. In this review, we will briefly discuss current knowledge of LLPCs, comparing mice and humans.

## Introduction

The bone marrow (BM) is one of the most important immunological organs in the body. Therein reside the hematopoietic stem cells that give rise to the cells in the blood including leukocytes. In the mid 1990's the BM was also suggested as the source of persistently high antigen-specific antibody titers (1). Not long after were the antibody secreting cells in the BM described. They were named plasma cells (PCs) and were shown to have a lifespan similar to long-lived memory B cells, i.e., long-lived PCs (LLPC) (2). Currently the general consensus is that LLPCs live for a (very) long time.

The cells that give rise to antibody-secreting cells are B cells, which develop from hematopoietic stem cells via precursors in the BM. As immature B cells they leave the BM and migrate via blood to the spleen where they mature into naïve B cells. B cells also circulate via blood to other secondary lymphoid organs, particularly to lymph nodes and gut-associated lymphoid tissues (GALT). B cells express a B cell antigen receptor (BCR) on their surface i.e., a membrane-bound antibody. When naïve B cells encounter their cognate antigen they become activated, and after clonal expansion differentiate into short- and/or long-lived antibody-secreting PCs. LLPCs are typically formed during the germinal center (GC) reaction. In the GCs, the B cells undergo clonal expansion and their BCRs are diversified by class switch recombination (CSR) and/or somatic hypermutation (SHM), where the former acts on the antibody constant region and the latter on the variable region. CSR results in the switching from IgM to IgG, IgA, or IgE and thereby a change in effector function, such as complement activation, while the antigen specificity is retained. SHM allows the BCRs to undergo affinity maturation, which results in cells that express BCRs with higher affinity to outcompete those with lower affinity (3). B cells that leave the GCs can join the pool of either memory B cells or PCs, where the PCs migrate to their survival nïche in organs such as the BM or gut where they can further differentiate into LLPCs.

As mentioned, PCs can be either short- or long-lived, and are terminally differentiated cells that do not proliferate further. Short-lived PCs are found in extrafollicular sites, such as the red pulp of the spleen and medullary chords of the lymph nodes, while LLPC are located in other organs such as the BM and GALT where they produce antibodies for long periods of time (4–6).

The different functions of PCs and naïve B cells are reflected in their different morphology. In contrast to naïve B cells, PCs have a larger cytoplasm with abundant rough endoplasmic reticulum and Golgi apparatus. These components are necessary for the production and secretion of large amounts of antibodies (7). Such high antibody production requires the cells to correct for protein misfolding and aggregation within the endoplasmic reticulum, and hence PCs constitutively activate the unfolded protein response. By contrast to naïve B cells, PCs no longer bind or present antigen, as these receptors—BCR and MHC Class II, respectively—are down regulated from the cell surface. This view might have to be reconsidered though, as it has recently been observed that the BCR remains on the cell surface and is functional in both IgA- and IgM-secreting PCs (8, 9).

## Long-lived plasma cells: a brief comparison between mice and men

### The adiposity of the bone marrow

#### Mice

Adipocytes are the cells that produce fat, and in mice the adiposity of the BM is low compared to humans (10). The amount of fat in the BM also differs between mouse strains (11). It has been suggested that there might be two types of BM adipose tissue (12), constitutive and regulated, where the former arises early in life while the latter forms later in life, increases with age and is distributed across the bone in a more scattered way. The distinction between these two adipose tissues is thus based on spatiotemporal distribution (13, 14).

#### Humans

In humans, there are two types of BM: red and yellow. The red BM is hematopoietically active and contains the platelets, red blood cells, and leukocytes. The yellow marrow mainly contains adipose tissue, which has an antagonistic effect on hematopoiesis (15). At birth the BM mostly contains red, hematopoietically active marrow. However, conversion from red to yellow marrow starts early in life. Around the age of 25 years, 50–70% of the BM has turned yellow and hematopoiesis is mainly restricted to the axial skeleton (ribs), proximal humerus (shoulder bone), and femur (thigh bone) (11, 16). It has also been suggested that, as in mice, there might be two types of adipose tissue in human BM (13).

The fact that mice have less adipose tissue and more red BM than humans suggests that a larger part of the BM is hematopoietically active during the lifetime of the mouse. However, whether less BM adipose tissue affects PC biology and long-term vaccine responses is still unclear. In this context, as rabbits start to accumulate adipocytes in the yellow marrow early in life (in adolescence) they might be a better model to decipher the effect of BM adipose tissues on human PC biology (17).

### Life expectancy and long-lived plasma cells

#### Mice

The life span for mice is 1–3 years, depending on the strain (18). In the mid 1990's it was demonstrated that, after infecting mice with lymphocytic choriomeningitis virus (LCMV), the initial antibody response occurred in the spleen. However, after clearing the initial infection, and for the remainder of the animal's life, the BM became the major site for long-term production of virus-specific antibodies (1). In another study, virus-specific PCs were adoptively transferred into naïve mice after depletion of memory B cells (by irradiation), and it was shown that a large fraction of the PCs in both the BM and spleen survived. These PCs produced antibodies for over a year in the absence of detectable memory B cells (19). Moreover, after immunization with ovalbumin, it was observed that the PCs were present in constant numbers in the BM and that they survived for more than 90 days without DNA synthesis (2). In yet another study, after oral immunization with ovalbumin and cholera toxin, antigen-specific LLPCs were detected in the GALT where they secreted IgA, and in the BM where they secreted IgG and IgA (20). At both sites, the antigen-specific LLPCs persisted for at least 9 months after immunization. This study also indicated crosstalk between the GALT and BM. Thus, in mice, LLPCs can survive for months or even a year or two. However, as the life span of mice is much shorter than that of humans, it becomes a hurdle when investigating the immunological persistence of, for instance, vaccine responses and LLPCs.

#### Humans

The life expectancy for humans in 2015 was 71.4 years (21). The persistence of antibody levels for life has been well-documented in humans (22, 23). Indeed, work performed in the late 1980's showed that the BM is a site of B cell development from precursor cells as well as for antibody-secreting cells (24–26). However, whether this is due to the longevity of LLPCs that are generated at the time of vaccination/disease or to spontaneous differentiation of antigen-specific memory B cells into “new” LLPCs is more difficult to address. Nevertheless, using a novel carbon dating technique a recent study showed that PCs can survive for up to 22 years in the GALT (27). Thus, investigating true LLPCs that survive for decades will require studies in human tissues.

### Cell surface markers

#### Mice

**Figure 1:**
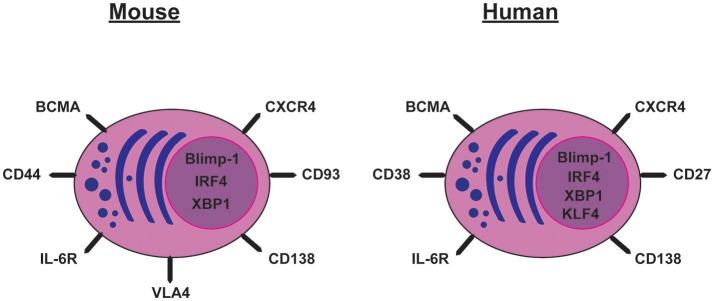
Cell surface markers and transcription factors expressed by mouse and human long-lived plasma cells.

B220 is a pan-B-lineage marker in mice though not B-lineage specific. In contrast, CD19 is specific to the B-lineage. Naïve and GC B cells express both of these markers on their cell surface whereas their expression was thought to be absent on PCs. However, B220 positive and negative PCs have been described, although it is currently unclear whether they are LLPCs (28). Further, PCs express CD138 (Syndecan-1), CD93, CD44, VLA-4, IL6-R, BCMA, and CXCR4 and, of these, CD138 is often used to distinguish LLPCs from other B-lineage cells in the BM (Figure 1) [reviewed in (29)].

#### Humans

Human PCs can be identified by their very high levels of both CD27 and CD38 and, as mouse PCs, they also express CD138 (30, 31). As in mice, they also express CXCR4, IL6-R, and BCMA. In the human BM, PCs that are CD19^+^ or CD19^−^ secreting vaccinia-specific antibodies have been described (32). The CD19^−^ PCs show characteristics similar to LLPCs in mice (30, 31) with a resistance to *in vivo* B cell depletion with Rituximab. Rituximab is an anti-CD20 antibody that targets most stages of B-cell maturation but not PCs, as they do not express CD20 (31). Although the CD19^−^ and CD19^+^ PCs show similar antibody heavy chain repertoires, the V_H_ mutation number and frequency vary depending on isotype (31). BM from infants aged 5–7 months lack CD19^−^ PCs (31), indicating that the CD19^+^ PCs appear earlier in life than the CD19^−^ PCs. The V_H_ repertoire of BM LLPCs is a mixture of PCs rather than being dominated by a large clonally-related population (30) and the heavy chain repertoire is stable for over 6.5 years (33). Thus, further work is still needed to understand how different phenotypes of LLPCs correlate to function in both humans and mice.

### Transcription factors

#### Mice

The gene expression pattern in PCs is distinct from that of activated B cells. For instance, the transcription factors Bcl-6, Pax5, and Bach2 are silenced in PCs whereas PC-specific genes are activated (34). One of the main regulators of PC differentiation is Blimp-1 (35–38), which is expressed in all PCs and some GC B cells that have a phenotype resembling PCs (35). Our understanding of e.g., Blimp-1 as a crucial factor for PC differentiation has benefited much from the introduction of reporter mice (Blimp-1 GFP) where the fate of PCs can be followed throughout the life of the mouse (39). Blimp-1 is required for full PC differentiation but the commitment to PC fate can be Blimp-1-independent (40). Many of the components of the unfolded protein response that are up-regulated in PCs are regulated by Blimp-1 (41). Together with Blimp-1, another transcription factor, IRF4, is responsible for terminating the transcriptional program of GC B cells, CSR, and promoting PC differentiation (42). Indeed, inactivation of IRF4 ablates PC formation (38). IRF4 also regulates XBP-1, which coordinates changes in the cellular structure and function of PCs (43) including maintaining Ig transcription (38). Blimp-1-deficient PCs lose the ability to secrete antibodies but retain their transcriptional identity, whereas XBP-1-deficient PCs show decreased antibody secretion (38).

Bcl-6 is a transcriptional repressor that is essential for GC formation and multiple other functions, such as proliferation and assessing DNA damage. Bcl-6 and Blimp-1 have a reciprocal relationship depending on the differentiation stage of the B cell. In general, B cells with high levels of Bcl-6 have a high proliferative capacity but low antibody secreting capacity while the converse is true for Blimp-1 (44, 45). Thus, PC differentiation and function depends on the presence of Blimp-1, IRF4, and XBP-1 and the absence of Bcl-6.

#### Humans

In humans, Blimp-1, IRF4, and XBP-1 are associated with commitment to the PC fate (35, 43). These and some of the other transcription factors mentioned above e.g., Bcl-6 might have the same role in humans as in mice. Recently, more factors involved in commitment to PC differentiation in humans have been discovered. For example, the transcription factor KLF4, which enhances the ability of plasmablasts to differentiate into PCs and LLPCs (46).

In conclusion, more work is still needed to understand LLPCs in humans but also mice. With the emergence of new techniques such as single cell RNA sequencing, more light will certainly be shed on the regulatory networks in both human and mouse LLPCs in the coming years.

### The survival nïche

IL-5 and IL-6 were among the earliest cytokines shown to have important roles in PC biology in both mice and humans (47–50). IL-5 was originally identified as a B-cell growth and differentiation factor (51) as well as an eosinophil differentiation and IgA-enhancing factor (52, 53). IL-6 was initially described in the early 1980s and named B-cell differentiation factor based on its ability to induce B cell differentiation (54, 55). In addition to these cytokines, other factors also contribute to PC survival, e.g., APRIL (see below).

#### Mice

Since the above-mentioned studies, it has been demonstrated that eosinophils and PCs co-localize in mouse BM and that eosinophils produce APRIL although also megakaryocytes and basophils can produce APRIL (56–58). It has also been shown that anti-IL-5 treatment decreases the expression of both APRIL and IL-6 mRNA in the BM (59). The exact role of IL-6 for LLPCs is still unclear since on one hand IL-6^−/−^ mice generate fewer Ag-specific PCs but on the other hand LLPCs transferred into an IL-6^−/−^ recipient mouse persist (49, 60). Although APRIL was already known to be important for PC survival, eosinophils were mainly known as important players in inflammatory responses related to allergies and immune defense against parasites (61–63) but not in the context of PCs. However, a recent report questions the importance of eosinophils for LLPCs in the BM since neither genetic deletion nor antibody treatment against eosinophils affected the LLPCs (64). In addition to hematopoeitic cells producing APRIL, osteoclasts might also contribute (65). Moreover, it has recently been shown that regulatory T cells are important for PC survival in the BM (66). It should also be mentioned that BAFF contributes to the survival of LLPCs (67) and, as BAFF is abundant in peripheral blood, under conditions such as inflammation CXCR4 positive LLPCs might leave the BM to reside in other CXCL12-rich tissues (68).

In mice, ~80% of all PCs and eosinophils are located in the GALT (69). Here, eosinophils are responsible for the generation and maintenance of IgA-secreting PCs and promotion of CSR to IgA, and they are also necessary for maintaining immune homeostasis in GALT (70).

Although LLPC are mostly considered to reside in the BM and GALT, a population of B220^−^CD138^+^ LLPCs that secrete IgM and persist for the lifetime in the spleen, but to a lesser extent in the BM, was recently described (71). This IgM-secreting LLPC population is distinct from BM resident LLPCs that mainly secrete IgG. The former develops in the absence of the GC reaction and shows no evidence of antigen selection. That is, the IgM from these LLPCs have a low level of somatic mutations, which contrasts the IgG-secreting PCs where mutations are abundant. The authors suggested that this process might be a pre-GC evolutionarily conserved pathway, such as the one protecting cartilaginous fish. Splenic LLPCs secreting mainly IgM have also been described in a mouse lupus model (NZW/B mice) where they survived for over 6 months (72).

#### Humans

A high number of eosinophils reside in the human gut and are in the vicinity of LLPCs (27). Gut eosinophils express high levels of APRIL and IL-6, which is thought to promote survival of PCs also in humans (70). Another report showed that IL-6 together with either APRIL or soluble factors from stromal cells are mandatory for the generation of LLPCs *in vitro* (73). It also appears that PCs prolong the survival of eosinophils in that secretory IgA binds to their FcαR receptor, which prevents apoptosis (74). However, the role of eosinophils in the survival of LLPCs in the human BM is less clear than in mice. There are indications, however, that eosinophils play a role in the persistence of high antibody titers in humans (75, 76).

Whether it is in the gut, BM, or spleen, the role of the environment appears to be one of the crucial factors for maintaining LLPCs and antibody titers. Elucidating the signals needed for their survival in different nïches and the role of other cell types such as osteoclasts and regulatory T cells require additional studies in the human setting.

### Plasma cells in disease

Generating high affinity antigen-specific antibodies is critical to protect against infections. However, on the other side of the coin are diseases related to PCs and production of autoantibodies in autoimmune diseases. Examples include anti-citrullinated protein antibodies in rheumatoid arthritis and anti-nuclear/anti-DNA antibodies in systemic lupus erythematous (SLE). There are also cancers of plasma cells (myelomas) that secrete mono/oligoclonal antibodies. A newly described systemic disorder is characterized by increased serum IgG4 levels with fibrotic changes in the affected area (77) and other organ-associated diseases (78–80).

### Plasma cells and the use of antibodies in the clinic

The immortalization of PCs to produce monoclonal antibodies (MAbs) resulted in a Nobel Prize in 1984 to George Köhler and Cesar Milstein (81). This has resulted in the widespread use of MAbs in diagnostics and in therapeutics targeting diseases. For instance, in 1997, anti-CD20 was the first MAb approved as a therapeutic to treat lymphoma. The anti-CD20 MAb has also been used in rheumatoid arthritis and cancers, including diffuse large B-cell lymphomas, melanoma, follicular lymphoma, and chronic lymphocytic leukemia but not LLPCs as they do not express CD20. In multiple myeloma, two other MAbs have been approved for treatment, anti-CD38 (Daratumumab) and anti-SLAMF7 (Elotuzumab) (82, 83). Both of these molecules are highly expressed on both healthy and malignant PCs and these two MAbs have turned out to work efficiently in multiple myeloma by NK cell mediated antibody-dependent cellular cytotoxicity (both) and complement dependent cytotoxicity as well as apoptosis via cross-linking (Daratumumab) (82, 83). There are also therapies directed against BAFF, a survival factor for B cells. For example, the human MAb Belimumab that antagonizes BAFF, which is approved as treatment for SLE and is under evaluation for treatment of severe active lupus nephritis in a phase III trial. Belimumab reduces the autoantibody levels and reduces the risk of severe disease flares (84). BAFF might also act directly on PCs, at least in disease situations, as therapies targeting BAFF are currently being tested in patients with multiple myeloma (85, 86). The known PC survival factor APRIL is also being targeted using the blocking agent Atacicept, which reduces autoantibody levels in patients with SLE (87). In addition, Mepolizumab, a MAb against IL-5, lowers eosinophil counts and reduces the number of asthma exacerbations in patients with eosinophilic asthma (88). Thus, the potential therapeutic targets for PCs are increasing based on our current understanding of regulatory pathways. For instance, the proteasome inhibitor Bortezomib is used to treat multiple myeloma effectively, which depletes both short-lived and LLPCs in SLE (89), and further investigations will likely uncover additional factors and mechanisms involved in the formation and regulation of LLPCs. In this context it is timely that the 2018 Nobel prize in Physiology or Medicine was just awarded to Allison and Honjo, for the discovery of checkpoint blockade therapy, which represents a new concept in cancer treatment, This depend on the treatment with MAbs but these do not to target the cancer cells directly but rather indirectly by releasing the breaks on T cells (anti-CTLA4 and anti-PD1) (90, 91).

In summary, there are already known important differences between mice and humans that need to be taken into account when considering results from studies of LLPCs, and additional studies are required to understand the impact of these differences on LLPC biology. Finally, in addition to the importance of PCs in mediating life-long protection to infectious diseases such as smallpox and measles, understanding the regulatory networks deciding PC fate will provide greatly needed possibilities to design new and more potent therapeutic strategies for diseases involving human LLPCs such as SLE, myelomas, and numerous others.

## Author contributions

LP, TO, and IR wrote the first draft. SB, M-JW, OG, and I-LM wrote a second modified draft. SB, OG, and I-LM wrote the final version of the manuscript.

### Conflict of interest statement

The authors declare that the research was conducted in the absence of any commercial or financial relationships that could be construed as a potential conflict of interest.
